# Evaluation of soybean [*Glycine max* (L.) Merr.] genotypes for yield, water use efficiency, and root traits

**DOI:** 10.1371/journal.pone.0212700

**Published:** 2019-02-22

**Authors:** Harrison Gregory Fried, Sruthi Narayanan, Benjamin Fallen

**Affiliations:** 1 Department of Plant and Environmental Sciences, Clemson University, Clemson, South Carolina, United States of America; 2 Pee Dee Research and Education Center, Clemson University, Florence, South Carolina, United States of America; Estacion Experimental del Zaidin, SPAIN

## Abstract

Drought stress has been identified as the major environmental factor limiting soybean [*Glycine max* (L.) Merr.] yield worldwide. Current breeding efforts in soybean largely focus on identifying genotypes with high seed yield and drought tolerance. Water use efficiency (WUE) that results in greater yield per unit rainfall is an important parameter in determining crop yields in many production systems, and is often related with crop drought tolerance. Even though roots are major plant organs that perceive and respond to drought stress, their utility in improving soybean yield and WUE under different environmental and management conditions are largely unclear. The objectives of this research was to evaluate soybean cultivars and breeding and germplasm lines for yield, WUE, root penetrability of hardpan, and root morphology. Field experiments were conducted at two locations in South Carolina (southeastern United States) during the 2017 cropping season to test the genotypes for yield and root morphology under irrigated and non-irrigated conditions. Two independent controlled-environmental experiments were conducted to test the genotypes for WUE and root penetrability of synthetic hardpans. The slow wilting lines NTCPR94-5157 and N09-13890 had equal or greater yield than the checks- cultivar NC-Raleigh and the elite South Carolina breeding line SC07-1518RR, under irrigated and non-irrigated conditions. The high yielding genotypes NTCPR94-5157, N09-13890, and SC07-1518RR exhibited root parsimony (reduced root development). This supported the recent hypothesis in literature that root parsimony would have adaptational advantage to improve yield under high input field conditions. The high yielding genotypes NTCPR94-5157, N09-13890, NC-Raleigh, and SC07-1518RR and a cultivar Boggs (intermediate in yield) possessed high WUE and had increased root penetrability of hardpans. These genotypes offer useful genetic materials for soybean breeding programs for improving yield, drought tolerance, and/or hardpan penetrability.

## Introduction

Soybean [*Glycine max* (L.) Merr.] is the most widely grown legume in the world and the fourth most important crop after wheat (*Triticum aestivum* L.), maize (*Zea mays* L.), and rice (*Oryza sativa* L.) in terms of area harvested and production [1]. It is the most important oil seed in the world with a contribution of > 60% to the total oil seed production and > 70% to the total protein meal consumption [2]. Currently, three countries—United States, Brazil, and Argentina- account for > 80% of the global soybean production [2]. Sustainability of soybean production in all soybean producing regions worldwide is threatened by climate change and associated environmental stresses [3, 4]. Drought stress has been identified as the major environmental factor limiting soybean yield in the United States and other regions of the world [4, 5, 6, 7, 8, 9, 10, 11].

Current breeding efforts in soybean largely focus on identifying genotypes with high seed yield and drought tolerance. Water use efficiency (WUE; the amount of biomass produced per unit water used) that results in greater yield per unit rainfall is an important parameter in determining crop yields in many production systems, and is often related with crop drought tolerance [12]. However, identification of high yielding crop cultivars with increased WUE is challenging because WUE associated with reduced water use often results in lower yield [13]. Increased WUE could be a useful selection criterion in crop breeding programs only if it is associated with high biomass and/or yield; a strategy to achieve that would be selecting for WUE based on increased biomass production rather than reduced water use [14, 15].

The physiological mechanisms underlying improved WUE in soybean under water- limited and non-limited conditions are not well understood. Roots might often be the first part of the plant that perceive drought stress and initiate response mechanisms [16]. The root distribution and architecture are critical in optimizing absorption of key resources such as water. Even though root traits that are associated with shoot traits contributing to productivity have been identified in soybean [17], their utility in breeding for yield improvement is yet to be determined. The difficulties associated with root harvest or evaluating root traits in situ (i.e., imaging living roots in soil) make root studies highly challenging [16, 18, 19]. Due to the lack of high throughput and cost-effective techniques for measuring root morphology and architecture under field conditions, ‘excavation’ still remains as the ‘gold standard’ for such measurements [16, 20, 21]. However, this technique is highly labor intensive. The unavailability of an efficient field-based methodology for root phenotyping has greatly impeded root studies in field crops. As a result, root system ideotypes that improve yield and/or WUE under different environmental and management conditions are largely unclear [10, 16, 22].

In the southeastern United States and many other soybean growing regions, the soybean crop is often grown on soils with a compacted zone or hardpan. Hardpans have high soil bulk density and they impose varying degrees of mechanical impedance to root growth. It limits root penetration and access to water and other soil resources. As a result, soybean plants become increasingly susceptible to water stress during periods of drought [10, 23, 24, 25]. So far, root penetrability of hardpan for improving yield and drought tolerance is a parameter of low priority in soybean breeding programs due to the lack of an efficient estimation method. Soybean genotypes with increased yield and WUE combined with hardpan penetrability can be good selections for many production regions including those that are prone to soil hardpan formation.

In a previous research, the current authors evaluated a diverse soybean germplasm collection of 49 genotypes for root morphological traits under controlled environmental conditions [17]. Based on the results, 10 genotypes were selected including cultivars, and breeding and germplasm lines that possessed varying root length, surface area, and volume (See Supplementary File 1 of Fried et al. [17]) for further analysis. The objective of this research was to evaluate the selected soybean genotypes for yield, WUE, root penetrability of hardpan, and root morphology. Yield of soybean genotypes was measured under field conditions. Water use efficiency and root penetrability of hardpan were measured under controlled environmental conditions. Root morphology was measured both under field conditions and controlled environmental conditions.

## Materials and methods

### Germplasm

The germplasm used in this study consisted of 10 soybean genotypes, out of which three were cultivars (Boggs, NC-Raleigh, and Crockett), one was a germplasm line (R01-581F) [26], and the rest were breeding lines (Table 1). A breeding line is an un-released genotype included in the breeding programs, which can be released as a germplasm line or a variety [27]. A breeding line gets released as a germplasm line if it has a promising trait(s), but does not have good agronomic performance, which is necessary to be released as a variety. The soybean genotypes used in this study belonged to maturity groups (MG) V, VI, VII, VIII (n = 1, 2, 5, and 2 respectively; recommended for south of latitude 28°N [28, 29]). The genotypes were selected based on their unique features, explained in the following. Genotypes N06-7023, N09-13890, and NTCPR94-5157 are slow wilting lines. Cultivar Boggs is intermediate in wilting. Genotype R01-581F has the ability to sustain nitrogen fixation under drought. Slow wilting (leaf wilting is delayed by several days, when soil dries) and sustained nitrogen fixation under drought conditions are two major traits associated with drought tolerance of soybean [26, 30, 31, 32, 33]. Two other genotypes included in this study (N09-12854 and SC-14-1127) are of exotic pedigree. Exotic germplasm has been found to be useful for improving yield and drought tolerance of soybean breeding populations in the United States [30, 34, 35]. A forage cultivar, Crockett was included in the study to test whether it’s increased aboveground vegetative growth is also associated with increased root growth. We included a conventional cultivar, NC-Raleigh and an elite South Carolina breeding line, SC07-1518RR in the study as checks, to serve as a comparison for the other genotypes in this study. Both NC-Raleigh and SC07-1518RR were developed for the production in the southeastern United States and have produced high yields in multiple regional variety tests [36, 37, 38].

**Table 1:** Characteristics of the soybean genotypes used in the study.

Genotype	Pedigree	Maturity group	Characteristics/Comments	Source of information	Geographical Origin
R01-581F	Jackson x KS 4895	V	Sustained nitrogen fixation under drought	[26]	AR, United States
Boggs	G81-152 x Coker 6738	VI	Intermediate in wilting	[39]	GA, United States
N06-7023	N98-7265 x N98-7288	VI	Slow wilting	[40]	NC, United States
N09-12854	N7103 x PI408337-BB	VII	Exotic pedigree	[41]	NC, United States
N09-13890	TCPR-83 x 11136	VII	Slow wilting (Pedigree traces back to a slow wilting line, PI 471938)	[32] Prior research of authors (unpublished data)	NC, United States
NC-Raleigh	N85-492 x N88-480	VII	Conventional cultivar -Check	[36]	NC, United States
NTCPR94-5157	Davis x N73-1102	VII	Slow wilting	[39]	NC, United States
SC-14-1127	NC Raleigh x PI 378696B (*Glycine soja*)	VII	Exotic pedigree	[42]	SC, United States
Crockett	PI 171451 x Hampton 266	VIII	Forage	[42, 43]	TX, United States
SC07-1518RR	SC01-809RR x G99-3211	VIII	Elite South Carolina breeding line—Check	N/A	SC, United States

### Evaluation of yield, root morphology, and shoot weight of soybean genotypes under field conditions

#### Plant husbandry

Field experiments were conducted at the Clemson University’s Pee Dee Research and Education Center, Florence, SC, USA [34°17'20.7"N, 79°44'18.4"W and 45.1 m above sea level (a.s.l.)] and Simpson Research and Education Center, Pendleton, SC, USA (34°38'51.4"N, 82°43'41.1"W and 260 m a.s.l.) during the 2017 cropping season (June to December at Florence and June to November at Pendleton). Both Florence (located in the south-eastern part of SC) and Pendleton (located in the northern part of SC) represent major soybean producing areas in the state. The characteristics of the experimental sites and field operations are given in Table 2. Soil tests were conducted before the commencement of the experiments, and based on the results, fertlizers were applied at both locations (Table 2). Weeds were controlled through pre- and post-emergent application of herbicides at both locations (Table 2). In addition, hand-weeding was performed whenever needed, at both locations. Soybean genotypes were planted in 4-row plots at both locations (details are given Table 2). At Florence, irrigation was provided during the vegetative and flowering stages. This consisted of 25.4 mm water applied at 35, 56, 69, 76, and 83 days after planting (DAP). Plants were at the early/mid flowering stage, depending up on the MG, by 76 DAP. At Pendleton, irrigation consisted of 25.4 mm water applied at 102 DAP during the late flowering/early pod formation stage (depending up on the MG). Due to the inaccessibility to the irrigation system, we could irrigate only once at Pendleton. Other details of irrigation are given in Table 2. No pest or pathogen problems were observed at both locations for the duration of the cropping season.

**Table 2:** The characteristics of the experimental sites and field operations at Florence, SC, USA and Pendleton, SC, USA.

Characteristics	Florence, SC	Pendleton, SC
Soil type	Norfolk sandy loam (fine-loamy, siliceous, thermic Typic Kandiudults)	Cecil sandy loam (clayey, kaolinitic, thermic Typic Hapludults)
Previous crops for the experimental site	Corn in 2016 and soybean in 2015	Sorghum in 2016 and soybean in 2015
Tillage	Primary tillage using a disk plow one week before planting	Primary tillage using a disk plow two weeks before planting
Fertilizer application	1. 0-0-60 (N-P-KCl) at the rate of 219 kg ha^-1^ 2. Dolomitic lime (CaMg(CO_3_)_2_) at the rate of 764 kg ha^-1^	1. 7-24-29 (N-P_2_O_5_-K_2_O) at the rate of 448 kg ha^-1^
Pre-emergent herbicide application	1. Valor (2-[7-fluoro-3,4-dihydro-3-oxo-4-(2-propynyl)- 2H-1,4-benzoxazin-6-yl]-4,5,6,7-tetrahydro-1H- isoindole-1,3(2H)-dione) (Valent USA, Snellville, GA) at the rate of 0.22 L ha^–1^ 2. Roundup (Glyphosate, N-(phosphonomethyl)glycine) (Monsanto, St. Louis, MO) at the rate of 2.34 L ha^–1^	1. Boundary (S-Metolachlor and Metribuzin) (Syngenta, Basil, Switzerland) at the rate of 1.75 L ha^–1^ 2. Prowl H_2_O (N-(1-ethylpropyl)-3,4-dimethyl-2,6-dinitrobenzenamine) (BASF Ag Products, Research Triangle Park, NC) at the rate of 2.33 L ha^–1^
Post-emergent herbicide application	1. Anthem Maxx (Pyroxasulfone) (FMC Agricultural Solutions, Philadelphia, PA) at the rate of 0.58 L ha^–1^ 2. Marvel (Fluthiacet methyl and Fomesafen) (FMC Agricultural Solutions, Philadelphia, PA) at the rate of 0.44 L ha^–1^.	1. Dawn (5-[2-chloro-4-(trifluoromethyl)phenoxy]-N-(methylsulfonyl)-2-nitrobenzamide) (Cheminova, Research Triangle Park, NC) at the rate of 4.68 L ha^–1^
Plot size^†^	6.1 m by 3.0 m	6.1 m by 3.0 m
Planting date	9 June 2017	8 June 2017
Planting depth	4 cm	4 cm
Type of planter	4-row dynamic disc planter (Wintersteiger, Salt Lake City, UT)	4-row cone planter (Almaco, Nevada, IA)
Row direction	North-south	North-south
Seeding rate	135,000 seeds ha^–1^	135,000 seeds ha^–1^
Row spacing	76.2 cm	76.2 cm
Irrigation	25.4 mm water applied at 35, 56, 69, 76, and 83 days after planting using a fixed-solid set sprinkler system	25.4 mm water applied at 102 days after planting using a travelling gun sprinkler system

^†^ All genotypes were sown in plots of 6.1 m by 3.0 m size in both locations

#### Experimental design

The field experiments were conducted using a split plot design with irrigation as the whole plot factor (two levels- irrigation and no-irrigation) and genotype as the sub plot factor (ten levels- ten different genotypes). The irrigation and genotype combinations were arranged in a 2x10 factorial treatment design. All treatments had five replications.

#### Dara collection

Root traits were measured at physiological maturity (growth stage R7) of plants in the field experiment at Florence. To measure root traits, root systems were excavated at 131 DAP for genotypes belonging to MG V and VI and at 157 DAP for genotypes belonging to MG VII and VIII. Root systems were excavated using a backhoe. The backhoe bucket excavated 121 cm x 64 cm x 45 cm (length x width x depth) of soil from the middle of the second row of each plot, which included root systems of 15–20 plants. Among them, five intact root systems were randomly chosen (subsamples), and were carefully separated from the soil with minimal root loss. At that point, the root systems, which were still attached to the shoot were gently shaken to remove soil adhering to them. After that, plants were cut at the base to separate shoots from the roots. Shoots were packed in paper bags and dried to constant weight at 70°C for determining dry weight. Shoot dry weight was expressed as weight per unit area (g m^-2^), which was estimated for each plot by dividing the shoot dry weight of the five plants sampled from that plot by the area occupied by them [area occupied by five plants was calculated using the number of plants per row, row length (6.1 m), and row spacing (76.2 cm)]. Root processing and analysis followed the protocol given by Fried et al. [17]. Briefly, root system of each plant was washed, placed between wet paper towels, sealed in Ziploc bags (S.C. Johnson & Sons, Inc. Racine, WI), and stored at 4°C. For further root analysis, roots were scanned using an Epson Perfection V600 scanner (6400 dpi resolution) (Epson, Long Beach, CA). To prepare root samples for scanning, the roots were taken out of the Ziploc bags, large root systems were cut in to smaller sections whenever necessary, and submerged in water within a tray (25 cm x 20 cm x 2 cm). This was to maximize separation and minimize overlap of roots. The root systems, including the nodules, were scanned while submerged in water in the tray. The scanned images of roots were analyzed using WinRHIZO Pro image analysis system (Regent Instruments, Inc., Quebec City, QC) to estimate the total root length (sum of the lengths of all roots in the root system), total root surface area, total root volume, fine root (diameter < 0.25 mm) length, fine root surface area, and fine root volume. These root traits were measured for each of the five plants sampled per plot, and the average value was estimated for each genotype in each plot. After scanning, roots were dried to constant weight at 70°C for determining dry weight. Root dry weight was expressed as weight per unit area (g m^-2^), which was estimated for each plot by dividing the root dry weight of the five plants sampled from that plot by the area occupied by them [area occupied by five plants was calculated using the number of plants per row, row length (6.1 m), and row spacing (76.2 cm)].

At harvest maturity (growth stage R8), plants were harvested for measuring seed yield. At both locations, the harvest dates were determined based on the growth stage of the plants, suitability of environmental conditions for harvest, and the availability of the combine-harvester. All plants from the middle two rows (second and third rows) of each plot were harvested at Pendleton, whereas all plants from the third and fourth rows were harvested at Florence (as some plants from the second row were already harvested for measuring root traits). At Pendleton, genotypes belonging to MG V and VI were harvested on 1 November 2017 (146 DAP) and genotypes belonging to MG VII and VIII were harvested on 16 November 2017 (161 DAP) using an Almaco SPC 20 combine (Almaco, Nevada, IA). At Florence, plants from MG V and VI were harvested by hand on 27 November 2017 (171 DAP). Hand-harvest was practiced due to unfavorable soil conditions for the use of a combine-harvester. The harvested plants were tied and stored in bundles in a dry storage room to prevent any shattering or damage, until they were taken for threshing. Genotypes belonging to maturity groups VII and VIII were harvested using an Almaco SPC-20 combine on 14 December 2017 (188 DAP) at Florence. Harvest was delayed due to wet environmental conditions. On the same day (14 December 2017), the hand harvested plants belonging to MG V and VI were threshed using the same Almaco SPC-20 combine. At both locations, seed yield (kg m^-2^) was calculated for each plot by dividing the fresh weight of the seeds harvested from two rows of that plot by the area occupied by those rows [area occupied by a row was calculated using the row length (6.1 m) and row spacing (76.2 cm)]. The seed moisture contents of genotypes were between 13 and 15% at harvest.

### Evaluation of water use efficiency and root traits of soybean genotypes under controlled environmental conditions

#### Plant husbandry

**Fig 1:**
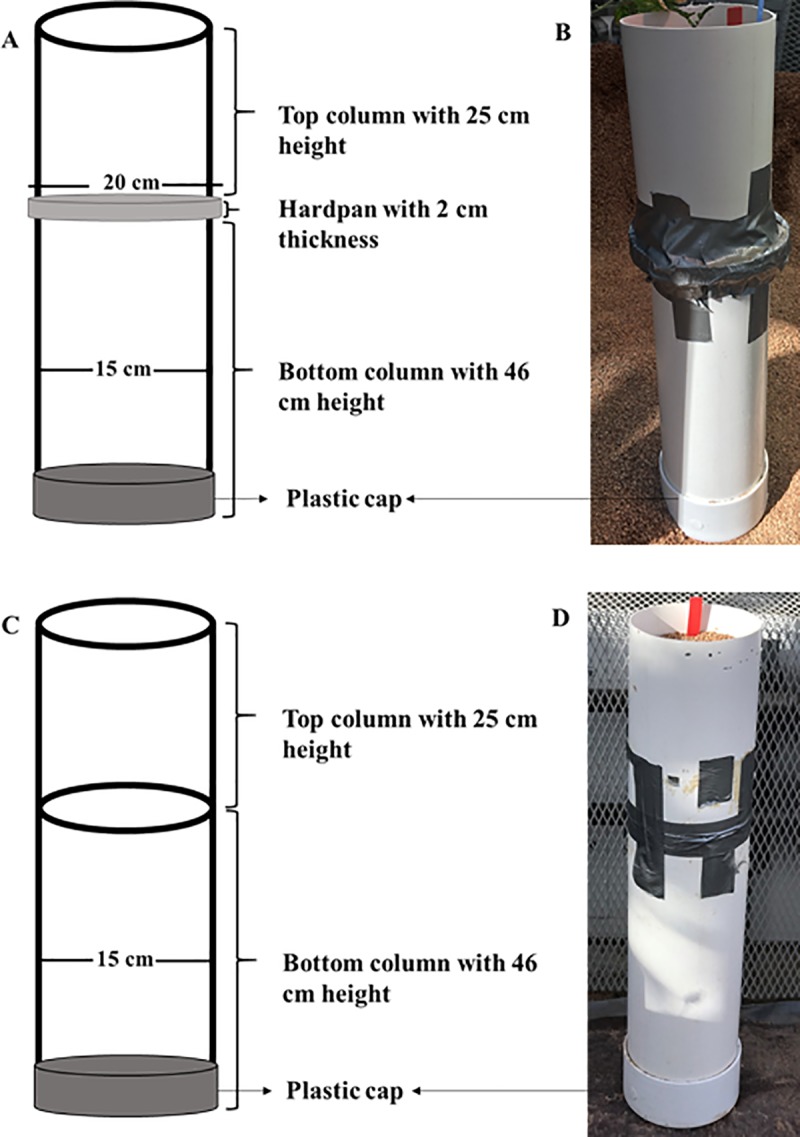
The mesocosms used to grow soybean plants in the experiment. Mesocosms were constructed of two stacked polyvinyl chloride (PVC) columns with an inside diameter of 15 cm. The height of the bottom and top columns were 46 and 25 cm, respectively. Each mesocosm was sealed at the bottom with a plastic cap, which had a central hole of 0.5 cm diameter for drainage. A diagram and a photograph of a mesocosm that contained a synthetic hardpan in between the top and bottom columns (A and B, respectively). The synthetic hardpan was made up of paraffin wax and petroleum jelly, and had a diameter of 20 cm and thickness of 2 cm. A diagram and a photograph of a mesocosm that did not contain a synthetic hardpan (C and D, respectively). The top and bottom columns along with the synthetic hardpan in between (if the mesocosm contained one) were tightly sealed together with a duct tape as shown in Fig 1B and 1D.

Two independent experiments (Run 1 and 2) were conducted to examine the WUE and root traits of the soybean genotypes under controlled environmental conditions in a greenhouse at Clemson, SC, USA (34°40'41.82"N, 82°50'21.03"W). The plant husbandry followed the methods given by Fried et al. [17]. The methods are briefed below with any modifications described in detail. The soybean plants were grown in mesocosms constructed of two stacked polyvinyl chloride (PVC) columns sealed at the bottom with a plastic cap (Fig 1). The bottom and top columns were of 46 and 25 cm height, respectively, with an inside diameter of 15 cm. The bottom column was filled with saturated Turface MVP (Burnett Athletics, Campobello, SC). Turface is calcined, non-swelling illite and silica clay, and is an efficient planting medium for root studies as described by Fried et al. [17] and Narayanan et al. [44, 45]. We wanted to examine the WUE of soybean genotypes when their root systems incur the stress resulting from a hardpan and in the absence of that. Therefore, in half of the mesocosms, a synthetic hardpan was placed on top of the bottom column. The synthetic hardpan was made up of 85% wax (Royal Oak Enterprises LLC, Roswell, GA) and 15% petroleum jelly (Vaseline; Unilever, Englewood Cliffs, NJ) by weight, and had a diameter of 16.5 cm, thickness of 1 cm, and strength (penetration resistance) of 1.5 MPa at 30°C (see supplementary figure 1 of Fried et al. [17] for more information). The wax-petroleum jelly system is an efficient approach to measure the penetrability of roots as described by previous researchers in several field crops including soybean [17, 46, 47, 48, 49, 50, 51, 52, 53]. The top column was placed on top of the wax-petroleum jelly synthetic hardpan (in half of the mesocosms that contained a synthetic hardpan) or directly on top of the bottom column (in the other half of the mesocosms that did not contain a synthetic hardpan). In this way, the synthetic hardpan was imposed at 25 cm depth in half of the mesocosms. The top and bottom columns along with the synthetic hardpan in between (if the mesocosm contained one) were tightly sealed together with a duct tape. After that, the top column was filled with saturated turface, which was later fertilized with a controlled-release fertilizer, Osmocote with 18:6:12, N:P_2_O_5_:K_2_O (Scotts, Marysville, OH) at a rate of 20 g per column before planting. To control sucking pests, such as aphids (*Aphis glycines* Matsumura), thrips [*Neohydatothrips variabilis* (Beach) and *Frankliniella spp*.], and whiteflies (*Bemisia tabaci*), a systemic insecticide, Marathon (a.i.: Imidacloprid: 1–[(6–Chloro–3–pyridinyl)methyl]–N–nitro–2–imidazolidinimine; OHP, Inc., Mainland, PA) was applied to the top column (1.7 g per column) before planting. Three seeds of a single genotype were sown in each column at a depth of 4 cm on 16 March 2018 for Run 1, and 15 May 2018 for Run 2. Thinning was performed after emergence (10 DAP) by retaining only the healthiest plant out of the three in each column, and removing the other two. In this way, each genotype was grown in four mesocosms (replications) containing the hardpan and four other mesocosms containing no hardpan. After thinning, the top of each mesocosm was covered with aluminum foil to prevent evaporation [32, 54, 55]. A small slit was made in the aluminum foil to allow the soybean plant to grow through. Immediately after covering the top with aluminum foil, each mesocosm was weighed to record their initial weight, which was later used for the estimation of plant water use. Plants were maintained under optimum temperature conditions (30/20°C, daytime maximum/nighttime minimum) [17, 56] and at a photoperiod of 13 hours until harvest [57]. No pest problems were observed on the plants in both runs. Plants were never watered during the 40 d growth period. At harvest (40 DAP; plants were at the vegetative growth stage), each mesocosm was weighed to record the final weight, which was used for the estimation of plant water use. Soil water contents in each mesocosm at harvest (40 DAP) are given as supporting information (See the excel sheet, ‘Controlled Environment Data’ in S1 File). No visual symptoms of water stress were observed on any plant until harvest. During harvest, plants were cut at the base to separate shoots from the roots. Shoots were packed in paper bags and dried to constant weight at 70°C for determining dry weight.

#### Experimental design

The controlled environmental experiments were conducted using a split plot design with hardpan as the whole plot factor [two levels- presence and absence of a hardpan in the plant growth columns (mesocosms)] and genotype as the sub plot factor (ten levels, ten different genotypes). The hardpan and genotype combinations were arranged in a 2x10 factorial treatment design. All treatments had four replications.

#### Data collection

Root harvest, processing, and further analysis followed the protocol given by Fried et al. [17]. Roots from the top and bottom columns were harvested, processed, and analyzed separately. The total or fine root length, surface area, and volume for any root system was estimated as the sum of those measures in the top and bottom columns. Penetrated root length (PRL) (measured for the plants grown in the mesocosms containing a hardpan) was estimated as the ratio between the length of the roots in the bottom column (i.e., below the hardpan) and the total length of the roots in the top and bottom columns (i.e., above and below the hardpan). After scanning, roots from the top and bottom columns were packed together in paper bags and dried to constant weight at 70°C for determining dry weight.

Water used by the soybean plants during the growth season was estimated in order to determine their WUE. Plant water use in each mesocosm was calculated by subtracting the final weight of the mesocosm (taken at the time of harvest at 40 DAP) from its initial weight (taken when it was covered with aluminum foil at 10 DAP) [54, 55]. Plant WUE was estimated as the ratio of the shoot dry weight to the water used [54, 55].

### Statistical analyses

For both field and controlled environmental data, analysis of variance was performed using the GLIMMIX procedure in SAS (Version 9.4, SAS Institute). Separation of least square means was done using the LSD test. The probability threshold level (α) for statistical significance was set at 0.05. For the field data, genotype and irrigation were treated as fixed effects and replication nested within irrigation was treated as a random effect. For the controlled environmental data, run, any interactions involving run, replication nested within run and hardpan were treated as random effects.

## Results

### Environmental conditions during the cropping season at the field experimental sites

**Fig 2:**
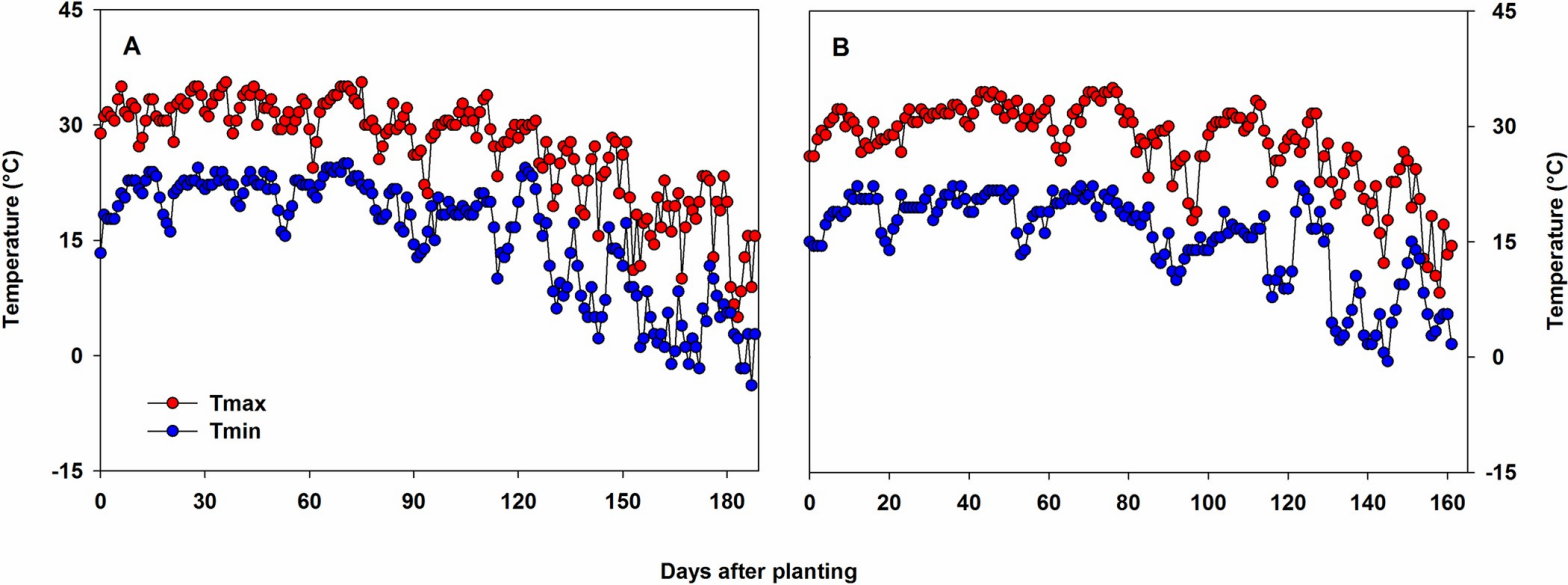
Daily maximum (Tmax) and minimum (Tmin) temperatures from planting through the end of the season at Florence, SC, USA (a) and Pendleton, SC, USA (b). Temperature data were obtained from the National Centers for Environmental Information (NCEI) of National Oceanic and Atmospheric Administration (NOAA). Soybean genotypes were planted on 9 June 2017 at Florence and 8 June 2017 at Pendleton. The duration of the crop season was 188 and 161 d at Florence and Pendleton, respectively.

**Fig 3:**
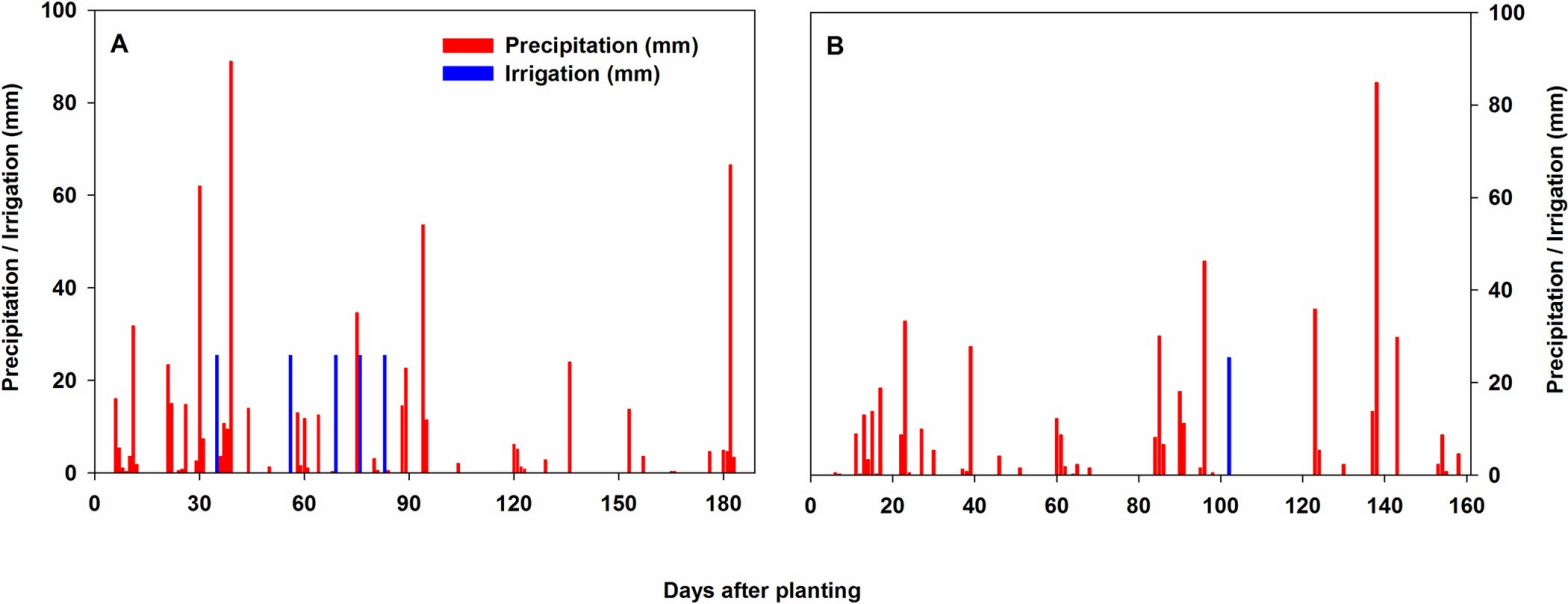
Daily precipitation and irrigation from planting through the end of the season at Florence, SC, USA (a) and Pendleton, SC, USA (b). Precipitation data were obtained from the National Centers for Environmental Information (NCEI) of National Oceanic and Atmospheric Administration (NOAA). Soybean genotypes were planted on 9 June 2017 at Florence and 8 June 2017 at Pendleton. The duration of the crop season was 188 and 161 d in Florence and Pendleton, respectively. Irrigation involved application of 25.4 mm water at 35, 56, 69, 76, and 83 days after planting (DAP) at Florence and application of 25.4 mm water at 102 DAP at Pendleton.

The cropping season spanned between 9 June 2017 and 14 December 2017 (188 d) at Florence and 8 June 2017 and 16 November 2017 (161 d) at Pendleton. The average maximum and minimum temperatures were 27°C and 16°C, respectively at Florence and 28°C and 15°C respectively at Pendleton (Fig 2). Total precipitation was 650 mm at Florence and 489 mm at Pendleton (Fig 3). The irrigated plots received a total of 127 mm and 25.4 mm of supplemented water at Florence and Pendleton, respectively.

### Yield, root morphology, and shoot weight of soybean genotypes under field conditions

**Fig 4:**
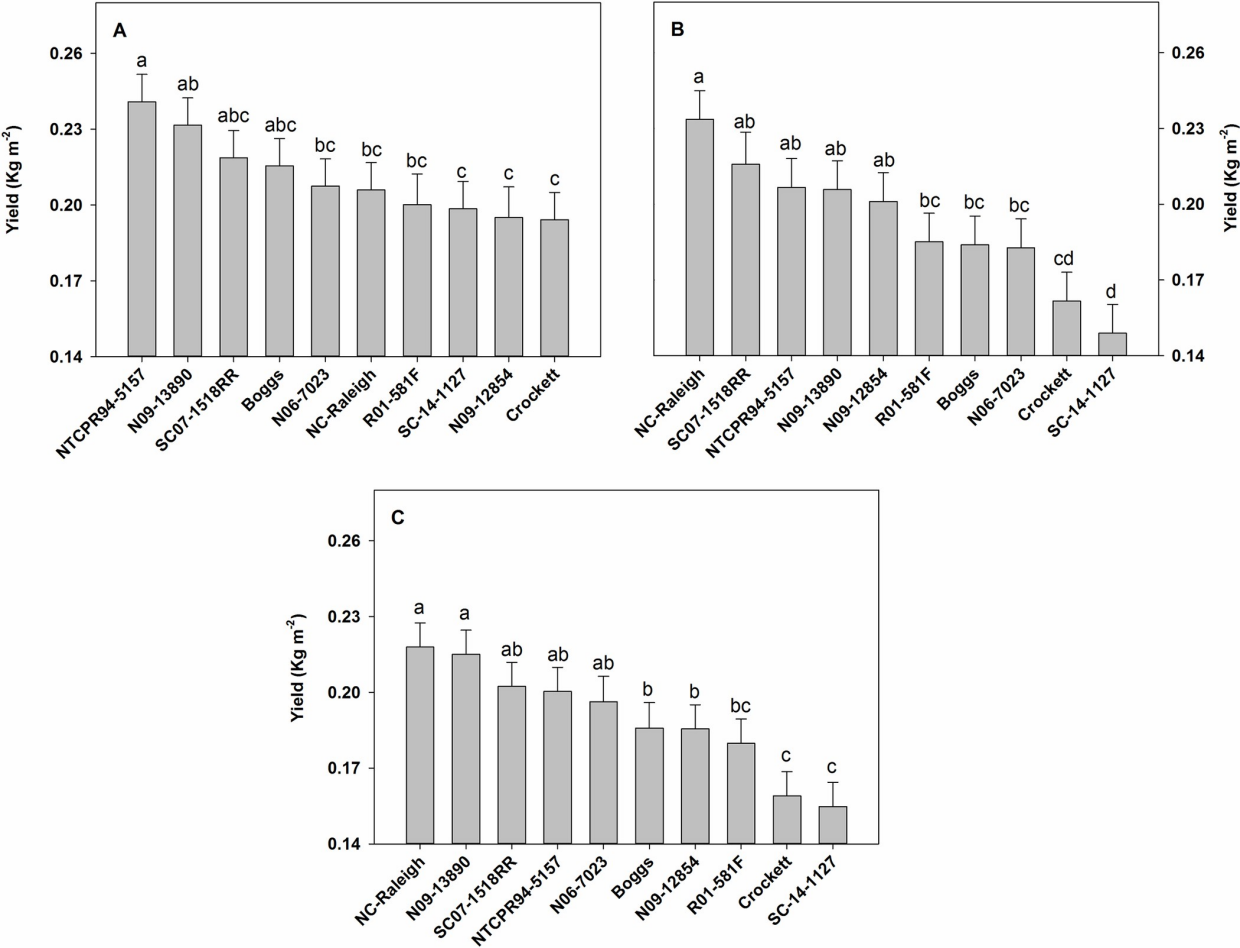
Seed yield of soybean genotypes grown at Florence, SC, USA under irrigated and non-irrigated conditions (Fig a and b, respectively) and at Pendleton, SC, USA (Fig c). Irrigated plots received 25.4 mm water at 35, 56, 69, 76, and 83 days after planting (DAP) at Florence and 25.4 mm water at 102 DAP at Pendleton. However, the genotype-by-irrigation interaction effect was not significant on yield at Pendleton. Therefore, data were averaged across irrigation treatments for this location. Bars represent least square means and error bars represent standard errors. Least square means with different letters are significantly different according to the LSD test at P < 0.05.

Genotype and irrigation had significant effects on seed yield of soybean genotypes at both locations (Table 3). However, the genotype-by-irrigation interaction effect was significant on yield only at Florence, where irrigation involved application of 25.4 mm water five times during the season between 35 and 83 DAP. The limited irrigation at Pendleton (25.4 mm water applied only once at 102 DAP) did not lead to a significant genotype-by-irrigation interaction effect on yield. Therefore, data presented in Fig 4 represent the genotype-by-irrigation interaction effect on yield at Florence and main effect of genotypes on yield at Pendleton. The elite South Carolina breeding line SC07-1518RR and two slow wilting lines NTCPR94-5157 and N09-13890 produced high yield at Pendleton and under both irrigated and non-irrigated conditions at Florence (Fig 4). The cultivar NC-Raleigh produced high yield at Pendleton and under non-irrigated conditions at Florence. It was intermediate in yield under irrigated conditions at Florence. The soybean cultivar Boggs (intermediate in wilting) and a slow wilting line N06-7023 were intermediate in yield at Pendleton and under both irrigated and non-irrigated conditions at Florence. A forage cultivar Crockett and a breeding line SC-14-1127 (exotic pedigree) were low yielders at Pendleton and under both irrigated and non-irrigated conditions at Florence. The breeding line SC-14-1127 (exotic pedigree) produced low and high yields under irrigated and non-irrigated conditions, respectively, at Florence and intermediate yields at Pendleton.

**Table 3:** Analysis of variance results on effects of genotype, irrigation, their interaction (for the field experiments), presence of hardpan, and it’s interaction with genotype (for the controlled environmental experiments) for various traits measured in the study. The field level measurements of root and shoot traits (total and fine root length, surface area, and volume, and shoot and root dry weights) were made only at one location (Florence, SC, USA).

Trait	Genotype	Irrigation^†^	Genotype x Irrigation	Hardpan^‡^	Genotype x Hardpan
Field conditions					
Yield^§^ (Florence, SC) (Kg m^-2^)	< .0001	0.0005	0.0449	N/A	N/A
Yield (Pendleton, SC) (Kg m^-2^)	< .0001	0.0074	0.9490	N/A	N/A
Total root length (cm)	0.0904	0.0413	0.9732	N/A	N/A
Total root surface area (cm^2^)	0.0013	0.1026	0.4274	N/A	N/A
Total root volume (cm^3^)	0.0322	0.0418	0.4229	N/A	N/A
Fine root^¶^ length (cm)	0.3205	0.0011	0.9897	N/A	N/A
Fine root surface area (cm^2^)	0.2864	0.0008	0.9939	N/A	N/A
Fine root volume (cm^3^)	0.2548	0.0006	0.9957	N/A	N/A
Shoot dry weight (g m^-2^)	0.0005	0.0473	0.7707	N/A	N/A
Root dry weight (g m^-2^)	0.2756	0.0214	0.1006	N/A	N/A
Controlled environmental conditions					
Total root length (cm)	0.0149	N/A	N/A	0.2690	0.5155
Total root surface area (cm^2^)	0.0050	N/A	N/A	0.2613	0.6925
Total root volume (cm^3^)	0.0055	N/A	N/A	0.1747	0.8554
Fine root length (cm)	0.0067	N/A	N/A	0.1628	0.4425
Fine root surface area (cm^2^)	0.0099	N/A	N/A	0.1863	0.4856
Fine root volume (cm^3^)	0.0136	N/A	N/A	0.2050	0.5196
Shoot dry weight (g)	0.0014	N/A	N/A	0.1236	0.2324
Root dry weight (g)	0.0029	N/A	N/A	0.1908	0.2460
Water use (kg)	0.2083	N/A	N/A	0.2575	0.6071
Water use efficiency^#^ (g kg^-1^)	0.0102	N/A	N/A	0.3460	0.5133

^†^At Florence, 25.4 mm water was applied at 35, 56, 69, 76, and 83 days after planting [DAP]. At Pendleton, 25.4 mm water was applied at 102 DAP.

^‡^In the controlled environmental experiments, a synthetic hardpan (1 cm thickness) that simulate a compacted soil layer was imposed at 25 cm depth in half of the plant growth columns to test the genotypes for water use efficiency under the presence and absence of a hardpan.

^§^Yield was measured at two locations (Florence and Pendleton) in SC, USA in 2017.

^¶^Diameter < 0.25 mm.

^#^Ratio between the amount of aboveground biomass produced and water used during a 40-day growth period.

Genetic variability was found for total root surface area, total root volume, and shoot dry weight under field conditions at Florence (Table 3). Data on root traits and shoot weight were collected only at that location. The genotype-by-irrigation interaction effect was not significant on any of the above root and shoot traits; therefore, the main effect of genotypes are presented in Table 4. Neither genotype nor genotype-by-irrigation interaction had significant effects on total root length, fine root length, fine root surface area, fine root volume, and root dry weight under field conditions (Table 3). Genotypes SC07-1518RR, NTCPR94-5157, and N09-13890 (high yields) had low total root surface area and volume under field conditions (Table 4). Among them, genotype SC07-1518RR had high shoot dry weight. The other two genotypes (NTCPR94-5157 and N09-13890) had low shoot dry weight. The cultivar NC-Raleigh had high total root surface area and volume, and shoot dry weight. Genotypes Crockett and SC-14-1127 (low yield) had high total root surface area and volume. Crockett also had high shoot weight, whereas SC-14-1127 had low shoot weight. The above results on the forage cultivar Crockett showed that the increased aboveground vegetative growth of this cultivar is also associated with increased root growth under field conditions.

**Table 4:** Total root surface area, total root volume, and shoot dry weight of soybean genotypes evaluated under field conditions in Florence, SC. Plants were grown under two irrigation treatments (irrigated and non-irrigated). Since the genotype-by-irrigation interaction effect was not significant on any of the below traits, main effects of genotype are presented.

Genotypes	Total root surface area (cm^2^)	Total root volume (cm^3^)	Shoot dry weight (g m^-2^)
R01-581F	357±45^cd^	8.45±2.3^bc^	585±53^cd^
Boggs	277±42^d^	5.90±2.17^c^	597±50^bcd^
N06-7023	381±42^cd^	8.01±2.17^bc^	727±50^abc^
N09-12854	548±45^a^	16.30±2.3^a^	568±53^d^
N09-13890	393±42^bcd^	8.87±2.17^bc^	571±50^d^
NC-Raleigh	470±42^abc^	12.71±2.17^ab^	815±50^a^
NTCPR94-5157	362±42^cd^	9.71±2.17^bc^	545±50^d^
SC-14-1127	438±42^abc^	13.39±2.17^ab^	587±50^cd^
Crockett	506±42^ab^	13.32±2.17^ab^	753±50^a^
SC07-1518RR	395±45^bcd^	8.54±2.3^bc^	738±53^a^

Values shown are least square means ± standard errors. Least square means with different letters are significantly different according to the LSD test at P < 0.05.

### Water use efficiency, hardpan penetrability, and root morphology of soybean genotypes under controlled environmental conditions

Significant genetic variability was observed for WUE, total and fine root length, surface area, and volume, and shoot and root dry weights under controlled environmental conditions (Table 3). The genotype-by-hardpan interaction effect was not significant on any of the above root and shoot traits; therefore, the main effect of genotypes is presented in Table 5. Genotypes did not differ in terms of water use (Table 3).

**Table 5:** Root and shoot traits of soybean genotypes evaluated under controlled environmental conditions. Plants were grown in growth columns, and a synthetic hardpan (1 cm thickness) that simulate a compacted soil layer was imposed at 25 cm depth in half of the columns to test the genotypes for root and shoot traits under the presence and absence of a hardpan. Since the genotype-by-hardpan interaction effect was not significant on any traits, main effects of genotype are presented below.

Genotypes	Total root length^†^ (cm)	Penetrated root length^‡^ (cm cm^-1^)	Total root surface area (cm^2^)	Total root volume (cm^3^)	Fine root^§^ length (cm)	Fine root surface area (cm^2^)	Fine root volume (cm^3^)	Shoot dry weight (g)	Root dry weight (g)	Water use efficiency^¶^ (g kg^-1^)
R01-581F	3750±1213^ab^	0.135±0.079^ab^	494±215^bc^	5.37±2.84^b^	1750±331^ab^	75.5±15.2^abc^	0.288±0.061^ab^	1.28±0.388^bc^	0.65±0.266^ab^	2.06±0.43^bc^
Boggs	3703±1213^ab^	0.121±0.079^ab^	475±215^bc^	4.96±2.84^b^	1803±331^ab^	77.9±15.2^ab^	0.297±0.061^a^	1.41±0.388a^bc^	0.57±0.266^bc^	2.20±0.43^ab^
N06-7023	2942±1213^c^	0.104±0.079^ab^	406±215^c^	5.02±2.84^b^	1302±331^c^	56.6±15.2^d^	0.217±0.061^c^	0.98±0.388^d^	0.54±0.266^c^	1.82±0.43^c^
N09-12854	3345±1215^bc^	0.031±0.079^b^	433±216^bc^	4.56±2.85^b^	1549±332^abc^	67.8±15.2^abcd^	0.261±0.061^abc^	1.17±0.389^cd^	0.60±0.266^abc^	1.92±0.43^bc^
N09-13890	4107±1213^a^	0.173±0.079^ab^	622±215^a^	7.79±2.84^a^	1778±331^ab^	76.6±15.2^ab^	0.292±0.061^a^	1.48±0.388^ab^	0.71±0.266^a^	2.13±0.43^abc^
NC-Raleigh	3369±1215^bc^	0.239±0.079^a^	500±216^bc^	6.07±2.85^b^	1391±332^c^	60.9±15.2^cd^	0.234±0.061^bc^	1.51±0.389^ab^	0.68±0.266^a^	2.44±0.43^a^
NTCPR94-5157	3934±1213^ab^	0.177±0.079^ab^	526±215^ab^	5.93±2.84^b^	1848±331^a^	79.2±15.2^a^	0.300±0.061^a^	1.28±0.388^bc^	0.66±0.266^ab^	2.01±0.43^bc^
SC-14-1127	3002±1213^c^	0.072±0.079^b^	435±215^bc^	5.37±2.84^b^	1358±331^c^	59.3±15.2^d^	0.228±0.061^c^	1.16±0.388^cd^	0.61±0.266^abc^	2.02±0.43^bc^
Crockett	3306±1213^bc^	0.041±0.079^b^	463±215^bc^	5.28±2.84^b^	1479±331^bc^	64.6±15.2^cd^	0.248±0.061^abc^	1.38±0.388^abc^	0.54±0.266^c^	2.10±0.43^abc^
SC07-1518RR	3540±1213^abc^	0.118±0.079^ab^	491±215^bc^	5.63±2.84^b^	1551±331^abc^	67.1±15.2^abcd^	0.256±0.061^abc^	1.57±0.388^abc^	0.70±0.266^a^	2.45±0.43^a^

Values shown are least square means ± standard errors. Least square means with different letters are significantly different according to the LSD test at P < 0.05.

^†^Sum of the lengths of all roots above and below the hardpan.

^‡^Ratio between length of the roots below the hardpan and the total length of the roots below and above the hardpan

^§^Diameter < 0.25 mm

^¶^Ratio between the amount of aboveground biomass produced and water used during a 40-day growth period.

Genotypes SC07-1518RR and N09-13890, which had high yield under field conditions also had high WUE, PRL, total and fine root length, surface area, and volume, and root and shoot dry weights under controlled environmental conditions (Table 5). Another high yielder under field conditions, NTCPR94-5157, was intermediate in terms of WUE and shoot dry weight, but had high PRL, total and fine root length and surface area, fine root volume, and root dry weight. The cultivar NC-Raleigh (high or intermediate yield) had high WUE, PRL, and shoot and root dry weights, but decreased length, surface area, and volume of total roots and fine roots. Similar to field conditions, Crockett (low yield) had high shoot dry weight under controlled environmental conditions. However, it was intermediate in terms of WUE and low in terms of all root traits. The relative performance of genotypes in terms of root length, surface area, and volume was generally consistent with that observed in the current authors’ earlier study under controlled environmental conditions (See Supplementary File 1 of Fried et al. [17]).

## Discussion

Current breeding strategies for soybean largely emphasize on drought tolerance as well as high seed yield. Since root morphological and anatomical traits are closely associated with whole-plant water acquisition, relevance and usefulness of these root traits in soybean breeding are gaining more importance in light of climate change and associated drought stress. However, high-throughput field-based root phenotyping techniques that allow simultaneous measurements of yield, WUE, and root traits are currently unavailable for field crops such as soybean [16, 58]. Therefore, this research that evaluated soybean cultivars and breeding and germplasm lines for yield, WUE, and root morphology provide valuable information to soybean breeding programs for yield improvement and drought tolerance.

The results from this research support the most recent hypothesis on ‘rightsizing’ the ‘hidden half’ of the plant for improving yield under high input agroecosystems [59]. This author proposed that a parsimonious root phenotype, which refers to reduced root development, would be advantageous for annual crops grown for seed yield in high input production systems that typically exist in many production regions all over the world. In the current high input crop production systems, application of fertilizers, herbicides, and pesticides and other crop management methods have reduced inter-plant competitions and minimized crop growth limitations such as inadequate availability of nutrients, especially nitrogen and phosphorous, root loss due to biotic stresses, root competition with weeds, and some abiotic stresses that are common in natural systems such as soil acidity [59, 60]. Thus, modern agronomic practices and crop breeding advancements have mitigated many constraints to root function that were prevalent in the agroecosystems in which crop ancestors evolved and crops were domesticated. However, a stress factor that still remains relevant and prevalent is drought. Thus, rather than the ancestral prolific root systems, the parsimonious root phenotypes that optimize water capture by reducing investments in cells, tissues, and organs with unfavorable cost/benefit ratio would be more advantageous in high-input production systems. Lynch [59] provided examples of parsimonious root architectural phenotypes as less number of axial roots, reduced lateral root density, reduced root plasticity to local resource availability, and greater loss of roots that do not improve water capture. Many of these characteristics directly influence root morphological traits such as length, surface area, and volume of the total roots and fine roots.

In the present study, the genotypes that produced high yields under irrigated and non-irrigated conditions (SC07-1518RR, NTCPR94-5157, and N09-13890) had less total root surface area and volume under field conditions (Table 4; Fig 4). At the same time, the genotypes that produced low yields (Crockett and SC-14-1127) had high total root surface area and volume under field conditions. Interestingly, genotypes SC07-1518RR, NTCPR94-5157, and N09-13890 (high yield under field conditions) had increased length, surface area, and volume of total roots and fine roots under controlled environmental conditions (Table 5). This implies that though these genotypes have the inherent ability to produce prolific root systems (characterized by high root length, surface area, and volume under controlled environmental conditions), when they were grown under high input field conditions, as an adaptation strategy, they might have partitioned less assimilates and energy to root systems and more to their reproductive tissues in order to increase seed yield. On the other hand, the production of prolific root systems (characterized by high root surface area and volume under field conditions) by genotypes Crockett and SC-14-1127 (low yield) might have acted as counterproductive by increasing intra-plant competition for assimilates and energy required for root growth as well as competition for the capture of mobile soil resources. The results from the present research are supported by previous research that found root parsimony as advantageous for yield and/or drought tolerance [60, 61, 62, 63, 64].

It is interesting to note that the slow wilting lines NTCPR94-5157 and N09-13890 had equal or greater yield than the checks- cultivar NC-Raleigh and the elite South Carolina breeding line SC07-1518RR, under irrigated and non-irrigated conditions at both locations. These genotypes also had the inherent potential to produce good root systems (characterized by high total and fine root length, surface area, and volume, PRL, and root dry weight under controlled environmental conditions), and were water use efficient. Their reduced total root surface area and volume under field conditions may be an example of root parsimony to improve yield under high input field conditions (Lynch hypothesis, see above). ‘Slow wilting’ is a largely used trait in the soybean breeding programs of the United States and many other production regions in the world for developing drought tolerant varieties [34]. The slow wilting nature of the genotypes NTCPR94-5157 and N09-13890 combined with their hardpan penetrability, root parsimony, and WUE make them wise selections for soybean breeding programs for variety development.

In the southeastern United States and many other production regions, most soils have an inherent compacted layer of subsoil (hardpan), which limits root penetration and crop yields. Farmers in these regions often practice expensive and non-sustainable tillage operations to increase the rooting zone. Since a viable approach to address this problem is to develop cultivars with root systems that penetrate the hardpan, the authors have started research to incorporate this trait into their soybean breeding programs. The present study found that the high yielding genotypes, SC07-1518RR, NTCPR94-5157, N09-13890, and NC-Raleigh had increased penetrability of hardpans (characterized by high PRL) (Table 5). A cultivar Boggs, which was intermediate in yield also had high PRL. Interestingly, the above five genotypes also had high WUE (Table 5). Taken together, these genotypes offer useful genetic materials for soybean breeding programs for hardpan forming regions.

Root growth and development under high input field conditions such as those existed in the present field study might be different from that under controlled environmental conditions when plants are grown in root columns. The present study captures these differences as it has measured root traits both under controlled environmental conditions and field conditions. However, WUE was only measured under controlled environmental conditions based on biomass production (WUE = biomass/water use). Field research is warranted to test how the genotypes will perform in terms of WUE based on seed yield under field conditions. Since suitable field-based techniques that allow simultaneous measurements of yield, WUE, and root traits are currently unavailable for field crops including soybean, and that has limited the knowledge generated in this area, the present research provide valuable information for soybean improvement.

## Conclusions

The present study supports the recent hypothesis in literature that root parsimony (reduced root development) would have adaptational advantage to improve crop yield under high input field conditions. We found that the slow wilting lines NTCPR94-5157 and N09-13890 had equal or greater yield than the checks- cultivar NC-Raleigh and the elite South Carolina breeding line SC07-1518RR, under irrigated and non-irrigated conditions. Interestingly, the high yielding genotypes NTCPR94-5157, N09-13890, and SC07-1518RR exhibited root parsimony (reduced root surface area and volume). In addition, the above four high yielding genotypes and a cultivar Boggs (intermediate in yield) also possessed high WUE and had increased ability to penetrate hardpans. These five genotypes offer useful genetic materials for soybean breeding programs for improving yield, drought tolerance, and/or hardpan penetrability.

## Supporting information

S1 File Excel file containing all data underlying the findings described in the paper. (XLSX)

S1 Table Root and shoot traits of soybean genotypes evaluated under controlled environmental conditions. Table below presents run 1 and run 2 results separately (runs indicate repeats of the study), whereas Table 5 in the paper presents results based on data pooled across runs [as there was no significant effect of ‘Run-by-Genotype-by-Hardpan’, ‘Run-by-Genotype’, and ‘Run-by-Hardpan’ on any of the traits we measured (P value > 0.05)]. In both runs, plants were grown in growth columns, and a synthetic hardpan (1 cm thickness) that simulate a compacted soil layer was imposed at 25 cm depth in half of the columns to test the genotypes for root and shoot traits under the presence and absence of a hardpan. Since the genotype-by-hardpan interaction effect was not significant on any traits in both runs, main effects of genotype are presented below. Values shown are least square means ± standard errors. Least square means with different letters are significantly different according to the LSD test at P < 0.05. Results were similar when data were analyzed across the runs (Table 5) or separately for runs 1 and 2 (Table below). This also indicates the reproducibility of results.
(DOCX)

## References

[1] FAO FAOSTAT; 2018 [cited 18 October 2018]. [Internet] Available at http://www.fao.org/faostat/en/#data/QC.

[2] SoyStats A reference guide to important soybean facts and figures. American Soybean Association. 2018 [cited 18 October 2018] [Internet] Available from: http://soystats.com/.

[3] Foyer CH, Lam HM, Nguyen HT, Siddique KH, Varshney RK, Colmer TD, et al Neglecting legumes has compromised global food and nutritional security. Nature Plants. 2016; 2: 16112 10.1038/nplants.2016.112 28221372

[4] Zipper SC, Qiu J, Kucharik CJ. Drought effects on US maize and soybean production: spatiotemporal patterns and historical changes. Environ. Res. Lett. 2016; 11: 094021

[5] Specht JE, Hume DJ, Kumudini SV. Soybean yield potential—a genetic and physiological perspective. Crop Sci. 1999; 39: 1560–1570.

[6] Oya T, Nepomuceno AL, Neumaier N, Farias JRB, Tobita S, Ito O. Drought tolerance characteristics of Brazilian soybean cultivars- evaluation and characterization of drought tolerance of various Brazilian soybean cultivars in the field. Plant Prod. Sci. 2004; 7: 129–137.

[7] Purcell LC, Specht JE. Physiological traits for ameliorating drought stress In: Boerma HR, and Specht JE, editors. Soybeans: improvement, production, and uses agronomy monograph 16. American Society of Agronomy, Crop Science Society of America, and Soil Science Society of America, Madison, Wisconsin, 2004 pp. 520–569.

[8] Battisti R, Sentelhas PC. Drought tolerance of Brazilian soybean cultivars simulated by a simple agrometeorological yield model. Expl Agric. 2015; 51: 285–298.

[9] Kunert KJ, Vorster BJ, Fenta BA, Kibido T, Dionisio G, Foyer CH. Drought stress responses in soybean roots and nodules. Front. Plant Sci. 2016; 7:1015 10.3389/fpls.2016.01015 27462339 PMC4941547

[10] Battisti R, Sentelhas PC. Improvement of soybean resilience to drought through deep root system in Brazil. Agron. J. 2017; 109: 1612–1622.

[11] Mertz-Henning LM, Ferreira LC, Henning FA, Mandarino JMG, Santos ED, Oliveira MCND, et al Effect of water deficit-induced at vegetative and reproductive stages on protein and oil content in soybean grains. Agronomy 2018; 8: 3.

[12] Richards RA, Rebetzke GJ, Condon AG, van Herwaarden AF. Breeding opportunities for increasing the efficiency of water use and crop yield in temperate cereals. Crop Sci. 2002; 42: 111–121. 11756261 10.2135/cropsci2002.1110

[13] Blum A. Drought resistance, water-use efficiency, and yield potential: Are they compatible, dissonant or mutually exclusive? Aust. J. Agric. Res. 2005; 56: 1159–1168.

[14] Specht JE, Chase K, Macrander M, Graef GL, Chung J, Markwell JP, et al Soybean response to water: a QTL analysis of drought tolerance. Crop Sci. 2001; 41: 493–509.

[15] Narayanan S, Aiken R, Prasad PVV, Xin Z, Yu J. Water and radiation use efficiencies in sorghum. Agron J. 2013; 105: 649–656.

[16] Fenta BA, Beebe SE, Kunert KJ, Burridge JD, Barlow KM, Lynch JP, et al Field phenotyping of soybean roots for drought stress tolerance. Agron J. 2014; 4: 418–435.

[17] Fried HG, Narayanan S, Fallen B (2018) Characterization of a soybean (*Glycine max* L. Merr.) germplasm collection for root traits. PLoS ONE 13(7): e0200463 10.1371/journal.pone.0200463. 29995945 PMC6040769

[18] Fitter AH. Characteristics and functions of root systems In: Waisel Y, Eshel A, Kafkafi U, editors. Plant roots: the hidden half. Marcel Dekker, New York, USA; 2002 pp. 249–259.

[19] Manschadi AM, Hammer GL, Christopher JT, deVoil P. Genotypic variation in seedling root architectural traits and implications for drought adaptation in wheat (*Triticum aestivum L*.). Plant Soil. 2008; 303: 115–129.

[20] Burridge J, Jochua CN, Bucksch A, Lynch JP. Legume shovelomics: High-throughput phenotyping of common bean (*Phaseolus vulgaris L*.) and cowpea (*Vigna unguiculata* subsp unguiculata) root architecture in the field. Field Crops Res. 2016; 192: 21–32.

[21] Trachsel S, Kaeppler SM, Brown KM, Lynch JP. Shovelomics: high throughput phenotyping of maize (*Zea mays* L.) root architecture in the field. Plant Soil. 2011; 341: 75–87.

[22] de Moraes MT, Bengough AG, Debiasi H, Franchini JC, Levien R, Schnepf A, et al Mechanistic framework to link root growth models with weather and soil physical properties, including example applications to soybean growth in Brazil. Plant Soil 2018; 428: 67–92.

[23] Busscher WJ, Lipiec J, Bauer PJ, Carter TE Jr. Improved root penetration of soil hard layers by a selected genotype. Commun. Soil Sci. Plant Anal. 2000; 31: 3089–3101.

[24] Nelson KA, Motavalli PP, Nathan M. Response of no-till soybean [*Glycine max* (L.) Merr.] to timing of preplant and foliar potassium applications in a claypan soil. Agron. J. 2005; 97: 832–838.

[25] Botta GF, Tolon-Becerra A, Lastra-Bravo X, Tourn M. Tillage and traffic effects (planters and tractors) on soil compaction and soybean (*Glycine max* L.) yields in Argentinean pampas. Soil Tillage Res. 2010; 110: 167–174.

[26] Chen P, Sneller CH, Purcell LC, Sinclair TR, King CA, Ishibashi T. Registration of soybean germplasm lines R01-416F and R01-581F for improved yield and nitrogen fixation under drought stress. J Plant Regist. 2007; 1: 166–167.

[27] Allard RW, Alvim PDe-T, Ashri A, Barton JH. Managing global genetic resources: The U.S. national plant germplasm system. National Academies Press, Washington, D.C 1991. 25144055

[28] Mourtzinis S, Conley SP. Delineating Soybean maturity groups across the United States. Agron. J. 2017; 109: 1397–1403.

[29] Scott WO, Aldrich SR. Modern Soybean Production. 1st Ed 1970 S & A Publ. Inc, Champaign, IL.

[30] Hufstetler EV, Boerma HR, Carter TE Jr, Earl HG. Genotypic variation for three physiological traits affecting drought tolerance in soybean. Crop Sci. 2007; 47: 25–35.

[31] King CA, Purcell LC, Brye KR. Differential wilting among soybean genotypes in response to water deficit. Crop Sci. 2009; 49: 290–298.

[32] Sadok W, Gilbert ME, Raza MA, Sinclair TR. Basis of slow-wilting phenotype in soybean PI 471938. Crop Sci. 2012; 52: 1261–1269.

[33] Sinclair TR, Purcell LC, King CA, Sneller CH, Chen P, Vadez V. Drought tolerance and yield increase of soybean resulting from improved symbiotic N_2_ fixation. Field Crops Res. 2007; 101: 68–71.

[34] Carter TE Jr, De Souza PI, Purcell LC. Recent advances in breeding for drought and aluminum resistance in soybean In: Kauffman H, editor. Proc. World Soybean Conf. VI Chicago, IL. 4–7 8 1999 Superior Print, Champaign, IL pp. 106–125.

[35] Lee GJ, Carter TE Jr, Boerma HR, Shannon JG, Hood M, Hawbaker M. Identification of soybean yield QTL in irrigated and rain-fed environments In Agronomy abstracts. ASA, Madison, WI 2002.

[36] Burton JW, Carter TE Jr, Fountain MO, Bowman DT. Registration of 'NC-Raleigh' soybean. Crop Sci. 2006; 46: 2710–2711.

[37] Soybean Official Variety Trials, Clemson University (2013, 2014, 2016, 2017) Available from https://www.clemson.edu/cafls/research/vt/soybeans.html. Accessed 18 October 2018.

[38] Soybean Official Variety Trials, University of Georgia (2013–2017) Available from http://www.swvt.uga.edu/ssfTests.html. Accessed 18 October 2018.

[39] Bellaloui N, Gillen AM, Mengistu A, Kebede H, Fisher DK, Smith Jr, et al Responses of nitrogen metabolism and seed nutrition to drought stress in soybean genotypes differing in slow-wilting phenotype. Front. Plant Sci. 2013; 4: 498 10.3389/fpls.2013.00498 24339829 PMC3857554

[40] SoyBase and the Soybean Breeder's Toolbox. [cited 18 October 2018] Database:Soybase [Internet] Available from: https://soybase.org/uniformtrial/index.php?filter=N06-7023&page=lines&test=ALL.

[41] Gillen AM, Shelton GW. Uniform soybean tests Southern states 2015. USDA -Agricultural Research Service Crop Genetics Research Unit 2015 Available from: https://www.ars.usda.gov/ARSUserFiles/60661000/UniformSoybeanTests/2015SoyBook.pdf.

[42] GRIN. Germplasm Resources Information Network. [cited 18 October 2018] Database:GRIN [Internet] Available from: https://www.ars-grin.gov/.

[43] Bowers GR Jr. Registration of 'Crockett' soybean. Crop Sci. 1990; 30: 427.

[44] Narayanan S, Mohan A, Gill KS, Prasad PVV. Variability of root traits in spring wheat germplasm. PLoS ONE. 2014a 9(6): e100317. 24945438 10.1371/journal.pone.0100317 PMC4063797

[45] Narayanan S, Prasad PVV. Characterization of a spring wheat association mapping panel for root traits. Agronomy Journal. 2014b; 106: 1593–1604.

[46] Acuña TLB, Pasuquin E, Wade LJ. Genotypic differences in root penetration ability of wheat through thin wax layers in contrasting water regimes and in the field. Plant Soil. 2007; 301: 135–149.

[47] Acuña TLB, Wade LJ. Root penetration ability of wheat through thin wax-layers under drought and well-watered conditions. Aust J Agric Res. 2005; 56: 1235–1244.

[48] Chimungu JG, Loades KW, Lynch JP. Root anatomical phenes predict root penetration ability and biomechanical properties in maize. J Exp Bot. 2015; 66: 3151–3162. 10.1093/jxb/erv121 25903914 PMC4449537

[49] Clark LJ, Aphale SL, Barraclough PB. Screening the ability of rice roots to overcome the mechanical impedance of wax layers: importance of test conditions and measurement criteria. Plant Soil. 2000; 219: 187–196.

[50] Clark LJ, Cope RE, Whalley WR, Barraclough PB, Wade LJ. Root penetration of strong soil in rainfed lowland rice: comparison of laboratory screens with field performance. Field Crops Res. 2002; 76: 189–198.

[51] Clark LJ, Price AH, Steele KA, Whalley WR. Evidence from near-isogenic lines that root penetration increases with root diameter and bending stiffness in rice. Funct Plant Biol. 2008; 35: 1163–1171. 10.1071/FP08132 32688863

[52] Yu LX, Ray JD, O’Toole JC, Nguyen HT. Use of wax-petrolatum layers for screening rice root penetration. Crop Sci. 1995; 35: 684–687.

[53] Zheng HG, Babu RC, Pathan MS, Ali L, Huang N, Courtois B, et al Quantitative trait loci for root-penetration ability and root thickness in rice: comparison of genetic backgrounds. Genome. 2000; 43: 53–61. 10701113

[54] Xin Z, Franks C, Payton P, Burke JJ. A simple method to determine transpiration efficiency in sorghum. Field Crops Res. 2008; 107: 180–183.

[55] Xin Z, Aiken R, Burke J. Genetic diversity of transpiration efficiency in sorghum. Field Crops Res. 2009; 111: 74–80.

[56] Djanaguiraman M, Prasad PVV, Schapaugh WT. High day- or nighttime temperature alters leaf assimilation, reproductive success, and phosphatidic acid of pollen grain in soybean [*Glycine max (L*.*) Merr*.]. Crop Sci. 2013; 53: 1594–1604.

[57] Bunce JA, Hilacondo WC. Responses of flowering time to elevated carbon dioxide among soybean photoperiod isolines. Am J Plant Sci. 2016; 7: 773–779.

[58] Zhu J, Ingram PA, Benfey PN, Elich T. From lab to field, new approaches to phenotyping root system architecture. Curr Opin Plant Biol. 2011; 14: 310–317. 10.1016/j.pbi.2011.03.020 21530367

[59] Lynch JP. Rightsizing root phenotypes for drought resistance. J Exp Bot. 2018; 69: 3279–3292. 10.1093/jxb/ery048 29471525

[60] Lynch JP. Steep, cheap and deep: an ideotype to optimize water and N acquisition by maize root systems. Ann Bot. 2013; 112: 347–357. 10.1093/aob/mcs293 23328767 PMC3698384

[61] Bolaños J, Edmeades GO, Martinez L. Eight cycles of selection for drought tolerance in lowland tropical maize. III. Responses in drought adaptive physiological and morphological traits. Field Crops Res. 1993; 31: 269–286.

[62] Gao Y, Lynch JP. Reduced crown root number improves water acquisition under water deficit stress in maize (*Zea mays L*.). J Exp Bot. 2016; 67: 4545–4557. 10.1093/jxb/erw243 27401910 PMC4973737

[63] Sebastian J, Yee MC, Viana WG, Rellán-Álvarez R, Feldman M, Priest HD, et al Grasses suppress shoot-borne roots to conserve water during drought. Proc Natl Acad Sci. 2016; 113: 8861–8866. 10.1073/pnas.1604021113 27422554 PMC4978293

[64] van Oosterom EJ, Zongjian Y, Zhang F, Deifel KS, Cooper M, Messina CD, et al Hybrid variation for root system efficiency in maize: potential links to drought adaptation. Funct Plant Biol. 2016; 43: 502–511. 10.1071/FP15308 32480480

