# Supplementary material for: Evaluation of soybean [Glycine max (L.) Merr.] genotypes for yield, water use efficiency, and root traits
Source: PLoS One. 2019 Feb 22;14(2):e0212700. doi: 10.1371/journal.pone.0212700 (PMC6386299; doi:10.1371/journal.pone.0212700)
Supplement: S1 Table — Table below presents run 1 and run 2 results separately (runs indicate repeats of the study), whereas Table 5 in the paper presents results based on data pooled across runs [as there was no significant effect of ‘Run-by-Genotype-by-Hardpan’, ‘Run-by-Genotype’, and ‘Run-by-Hardpan’ on any of the traits we measured (P value > 0.05)]. In both runs, plants were grown in growth columns, and a synthetic hardpan (1 cm thickness) that simulate a compacted soil layer was imposed at 25 cm depth in half of the columns to test the genotypes for root and shoot traits under the presence and absence of a hardpan. Since the genotype-by-hardpan interaction effect was not significant on any traits in both runs, main effects of genotype are presented below. Values shown are least square means ± standard errors. Least square means with different letters are significantly different according to the LSD test at P < 0.05. Results were similar when data were analyzed across the runs (Table 5) or separately for runs 1 and 2 (Table below). This also indicates the reproducibility of results. (DOCX) [file pone.0212700.s002.docx]

**S1 Table.** Root and shoot traits of soybean genotypes evaluated under controlled environmental conditions. Table below presents run 1 and run 2 results separately (runs indicate repeats of the study), whereas Table 5 in the paper presents results based on data pooled across runs [as there was no significant effect of ‘Run-by-Genotype-by-Hardpan’, ‘Run-by-Genotype’, and ‘Run-by-Hardpan’ on any of the traits we measured (P value > 0.05)]. In both runs, plants were grown in growth columns, and a synthetic hardpan (1 cm thickness) that simulate a compacted soil layer was imposed at 25 cm depth in half of the columns to test the genotypes for root and shoot traits under the presence and absence of a hardpan. Since the genotype-by-hardpan interaction effect was not significant on any traits in both runs, main effects of genotype are presented below. Values shown are least square means ± standard errors. Least square means with different letters are significantly different according to the LSD test at P < 0.05. Results were similar when data were analyzed across the runs (Table 5) or separately for runs 1 and 2 (Table below). This also indicates the reproducibility of results.

| Genotypes | Total root length^†^ (cm) | Penetrated root length^‡^ (cm cm^-1^) | Total root surface area (cm^2^) | Total root volume (cm^3^) | Fine root^§^ length (cm) | Fine root surface area (cm^2^) | Fine root volume (cm^3^) | Shoot dry weight (g) | Root dry weight (g) | Water use efficiency^¶^ (g kg^-1^) |
| --- | --- | --- | --- | --- | --- | --- | --- | --- | --- | --- |
| Run 1 | | | | | | | | | | |
| R01-581F | 2746±341^ab^ | 0±0.071^b^ | 301±38^ab^ | 2.67±0.51^a^ | 1572±201^a^ | 66.2±8.42^a^ | 0.247±0.031^a^ | 0.95±0.14^abc^ | 0.39±0.04^ab^ | 1.75±0.19^abc^ |
| Boggs | 2776±341^a^ | 0±0.071^b^ | 324±38^ab^ | 3.15±0.51^a^ | 1554±201^a^ | 66.3±8.42^a^ | 0.250±0.031^a^ | 1.00±0.14^abc^ | 0.35±0.04^abc^ | 1.69±0.19^abcd^ |
| N06-7023 | 1457±341^c^ | 0±0.071^b^ | 188±38^c^ | 2.99±0.51^a^ | 786±201^c^ | 33.9±8.42^c^ | 0.129±0.031^c^ | 0.53±0.14^d^ | 0.28±0.04^c^ | 1.22±0.19^d^ |
| N09-12854 | 2618±341^ab^ | 0 ±0.071^b^ | 288±38^abc^ | 2.55±0.51^a^ | 1476±201^ab^ | 63.3±8.42^ab^ | 0.239±0.031^ab^ | 0.99±0.14^abc^ | 0.39±0.04^ab^ | 1.63±0.19^abcd^ |
| N09-13890 | 2835±341^a^ | 0.108±0.071^b^ | 346±38^a^ | 3.43±0.51^a^ | 1524±201^a^ | 64.8±8.42^a^ | 0.244±0.031^a^ | 0.98±0.14^abc^ | 0.39±0.04^ab^ | 1.73±0.19^abcd^ |
| NC-Raleigh | 2342±341^abc^ | 0.333±0.071^a^ | 293±38^ab^ | 2.97±0.51^a^ | 1195±201^abc^ | 51.7±8.42^abc^ | 0.197±0.031^abc^ | 1.21±0.14^ab^ | 0.45±0.04^a^ | 2.10±0.19^ab^ |
| NTCPR94-5157 | 2295±341^abc^ | 0.117±0.071^b^ | 259±38^abc^ | 2.78±0.51^a^ | 1352±201^ab^ | 56.3±8.42^abc^ | 0.208±0.031^abc^ | 0.88±0.14^bcd^ | 0.38±0.04^abc^ | 1.60±0.19^bcd^ |
| SC-14-1127 | 1994±341^abc^ | 0.079±0.071^b^ | 256±38^abc^ | 3.13±0.51^a^ | 1071±201^abc^ | 46.4±8.42^abc^ | 0.177±0.031^abc^ | 0.75±0.14^cd^ | 0.34±0.04^bc^ | 1.44±0.19^cd^ |
| Crockett | 1863±341^bc^ | 0±0.071^b^ | 229±38^bc^ | 2.30±0.51^a^ | 982±201^bc^ | 42.1±8.42^bc^ | 0.159±0.031^bc^ | 0.88±0.14^bcd^ | 0.28±0.04^c^ | 1.74±0.19^abc^ |
| SC07-1518RR | 2167±341^abc^ | 0±0.071^b^ | 236±38^bc^ | 2.07±0.51^a^ | 1200±201^abc^ | 51.0±8.42^abc^ | 0.192±0.031^abc^ | 1.29±0.14^a^ | 0.39±0.04^ab^ | 2.12±0.19^a^ |
| Run 2 | | | | | | | | | | |
| R01-581F | 4754±379^abc^ | 0.270±0.091^a^ | 687±63^bcd^ | 8.08±0.91^bc^ | 1929±150^abc^ | 84.92±6.52^abc^ | 0.329±0.025^abc^ | 1.60±0.16^abc^ | 0.91±0.06^ab^ | 2.37±0.18^ab^ |
| Boggs | 4631±379^abc^ | 0.241±0.091^a^ | 626±63^bcd^ | 6.78±0.91^bc^ | 2051±150^ab^ | 89.50±6.52^ab^ | 0.344±0.025^ab^ | 1.83±0.16^ab^ | 0.79±0.06^b^ | 2.71±0.18^ab^ |
| N06-7023 | 4428±379^bc^ | 0.208±0.091^a^ | 623±63^bcd^ | 7.04±0.91^bc^ | 1819±150^bc^ | 79.37±6.52^bc^ | 0.305±0.025^bc^ | 1.43±0.16^bc^ | 0.80±0.06^b^ | 2.42±0.18^ab^ |
| N09-12854 | 3946±409^c^ | 0.062±0.091^a^ | 560±68^d^ | 6.35±0.98^c^ | 1554±162^c^ | 69.49±7.04^c^ | 0.272±0.028^bc^ | 1.29±0.17^c^ | 0.81±0.07^b^ | 2.20±0.19^b^ |
| N09-13890 | 5378±379^ab^ | 0.239±0.091^a^ | 898±63^a^ | 12.15±0.91^a^ | 2032±150^ab^ | 88.45±6.52^ab^ | 0.340±0.025^abc^ | 1.98±0.16^a^ | 1.03±0.06^a^ | 2.52±0.18^ab^ |
| NC-Raleigh | 4401±409^bc^ | 0.146±0.091^a^ | 714±68^bcd^ | 9.38±0.98^b^ | 1574±162^c^ | 69.48±7.04^c^ | 0.270±0.028^c^ | 1.82±0.17^ab^ | 0.90±0.07^ab^ | 2.76±0.19^a^ |
| NTCPR94-5157 | 5573±379^a^ | 0.238±0.091^a^ | 794±63^ab^ | 9.08±0.91^b^ | 2344±150^a^ | 102.06±6.52^a^ | 0.391±0.025^a^ | 1.68±0.16^abc^ | 0.95±0.06^ab^ | 2.42±0.18^ab^ |
| SC-14-1127 | 4011±379^c^ | 0.065±0.091^a^ | 614±63^cd^ | 7.61±0.91^bc^ | 1645±150^bc^ | 72.17±6.52^bc^ | 0.279±0.025^bc^ | 1.58±0.16^abc^ | 0.88±0.06^ab^ | 2.60±0.18^ab^ |
| Crockett | 4749±379^abc^ | 0.083±0.091^a^ | 698±63^bcd^ | 8.25±0.91^bc^ | 1977±150^abc^ | 87.06±6.52^abc^ | 0.337±0.025^abc^ | 1.88±0.16^ab^ | 0.80±0.06^b^ | 2.46±0.18^ab^ |
| SC07-1518RR | 4913±379^abc^ | 0.235±0.091^a^ | 746±63^abc^ | 9.19±0.91^b^ | 1901±150^bc^ | 83.07±6.52^bc^ | 0.320±0.025^bc^ | 1.85±0.16^ab^ | 1.01±0.06^a^ | 2.78±0.18^a^ |

^†^Sum of the lengths of all roots above and below the hardpan.

^‡^Ratio between length of the roots below the hardpan and the total length of the roots below and above the hardpan

^§^Diameter < 0.25 mm

^¶^Ratio between the amount of aboveground biomass produced and water used during a 40-day growth period.
